# Plasma ATG5 is increased in Alzheimer’s disease

**DOI:** 10.1038/s41598-019-41347-2

**Published:** 2019-03-18

**Authors:** Sun-Jung Cho, Hyun Joung Lim, Chulman Jo, Moon Ho Park, Changsu Han, Young Ho Koh

**Affiliations:** 1 Division of Brain Diseases, Center for Biomedical Sciences, Korea National Institute of Health, 187 Osongsaengmyeong2-ro, Osong-eup, Heungdeok-gu, Cheongju-si, Chungcheongbuk-do 28159 Korea; 2 Departments of Neurology, Korea University, Ansan Hospital, 123 Jeokgeum-ro, Danwon-gu, Ansan-si, Gyeonggi-do 15355 Korea; 3 Psychiatry, College of Medicine, Korea University, Ansan Hospital, 123 Jeokgeum-ro, Danwon-gu, Ansan-si, Gyeonggi-do 15355 Korea

## Abstract

Alzheimer’s disease (AD) is a major cause of dementia. Growing evidence suggests that dysregulation of autophagy, a cellular mechanism essential for self-digestion of damaged proteins and organelles, is involved in neurological degenerative diseases including AD. Previously, we reported that autophagosomes are increased in the brains of AD mouse model. However, the plasma levels of autophagic markers have not yet been investigated in patients with AD. In this study, we investigated the expression of autophagy-related genes 5 and 12 (ATG5 and ATG12, respectively) in cells *in vitro* upon amyloid-beta (Aβ) treatment and in the plasma of AD patients. ATG5-ATG12 complex levels were increased in primary rat cortical neurons and human umbilical vein endothelial cells after Aβ treatment. Furthermore, we compared plasma from 69 patients with dementia, 82 patients with mild cognitive impairment (MCI), and 127 cognitively normal control participants. Plasma levels of ATG5 were significantly elevated in patients with dementia (149.3 ± 7.5 ng/mL) or MCI (152.9 ± 6.9 ng/mL) compared with the control subjects (129.0 ± 4.1 ng/mL) (*p* = 0.034, *p* = 0.016, respectively). Our results indicate that alterations in the plasma ATG5 levels might be a potential biomarker in patients at risk for AD.

## Introduction

Alzheimer’s disease (AD) is the most common cause of dementia and is pathologically characterized by accumulation of amyloid-beta (Aβ) and intracellular neurofibrillary tangles in the brain. AD involves neuronal loss in the cerebral cortex and hippocampus leading to impaired cognitive functions^1^. The studies have reported that the endosomal-lysosomal pathway is involved in the processing of amyloid precursor protein (APP) to generate Aβ. Moreover, dysregulated endosomal-lysosomal pathway activity is observed in neurons affected by various stress factors. Recently accumulated evidence implicated that autophagy might be involved in neurodegenerative diseases such as AD and Parkinson’s disease.

Autophagy is an intracellular mechanism involved in the elimination and recycling of proteins and organelles by lysosomes^2^. Dysregulation of autophagy induces marked accumulation of autophagic vesicles (AVs) in affected neurons. Pathological evidence showed the accumulation of AVs in damaged neuritic processes and synaptic terminals are observed in AD^3^. Moreover, it is clear that dysfunction in the autophagy-lysosomal degradation likely precedes the formation of AD pathological hallmarks^4^. Genome-wide analysis in AD patients showed that positive regulators of autophagy are observed enriched in the entorhinal cortex, suggesting that autophagy-related genes are upregulated in the brains of AD patients^5^. However, *in vivo* evidence from patients implicating autophagy in AD pathology is still lacking and thus the role of autophagy in AD needs further investigation.

ATG5, encoded by autophagy-related gene 5 (*ATG5*), a key autophagy gene, is first conjugated to ATG12 by ATG7 and ATG10 and then promotes the lipidation of LC3^6^. Further, Aβ induces the conjugation of ATG5 and ATG12 in cells^5,7^. Beclin-1, ATG12, ATG5, and LC3 immunoreactivities were observed in neurons and endothelial cells in AD patients^8^. Moreover, recent studies have shown that increased plasma levels of autophagic markers might be associated with coronary total occlusion and childhood cerebral palsy^9,10^. Given that the increase in autophagic markers, such as Beclin-1 and LC3, is observed in the cerebrospinal fluid (CSF) and serum in patients with acute ischemic stroke^11^, they should be examined as potential fluid biomarkers. Despite the important role of autophagy in AD pathogenesis attributed *in vitro*, data from AD patients involving autophagy is insufficient. Moreover, the clinical relevance of ATG5 and ATG12 is still unknown.

Here, we investigated the proteins of the autophagic machinery, in particular ATG5 and ATG12 in AD patients as well as in cells *in vitro* upon Aβ treatment in order to examine the importance of these autophagic markers as potent biomarkers for AD.

## Results

### ATG5-ATG12 conjugation is induced in the endothelial cell-conditioned media upon Aβ treatment

**Figure 1:**
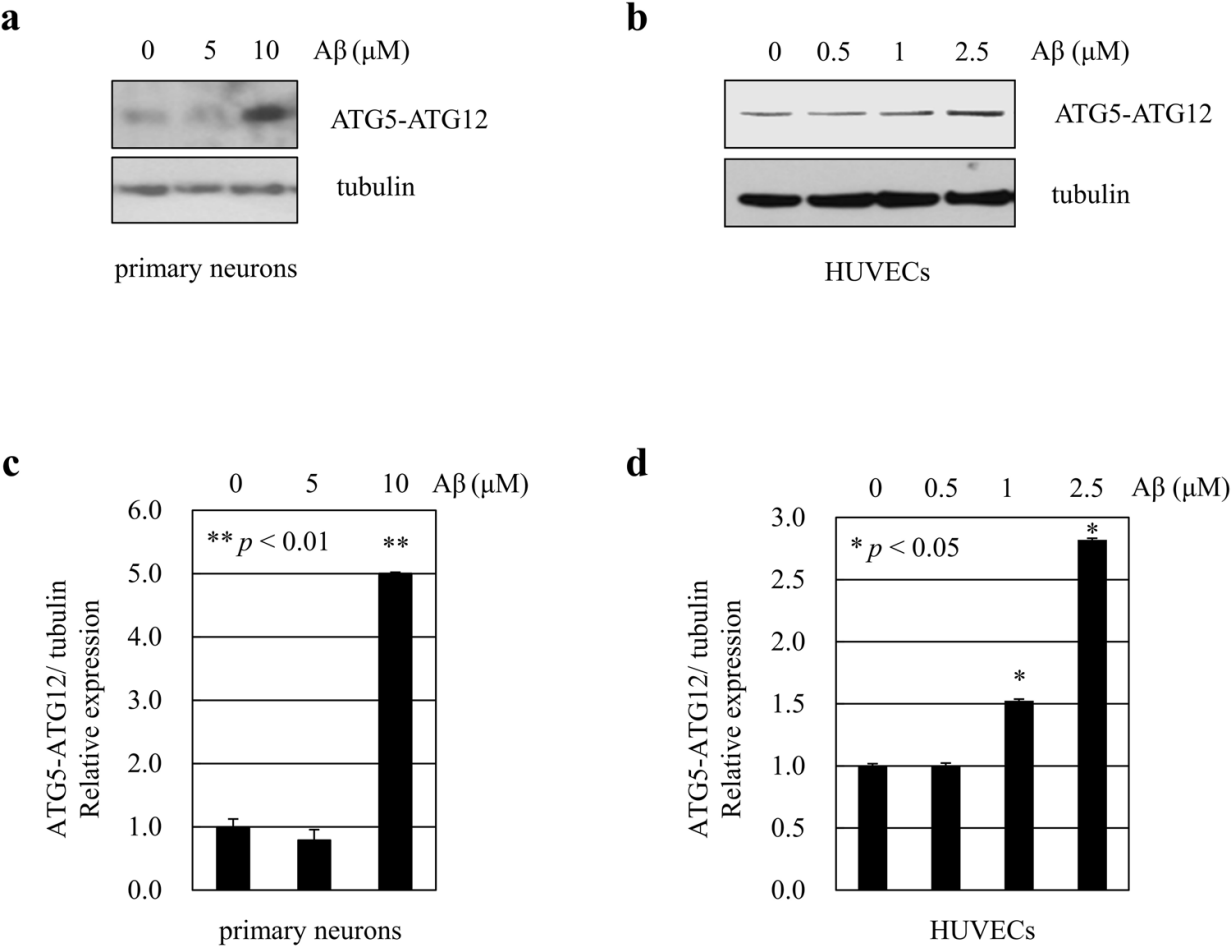
Aβ increases the level of conjugation of ATG5 and ATG12 in cells. (**a**) Primary neurons were treated with synthetic Aβ_1–40_ peptides. Forty-eight hours after treatment, Western blotting was performed with anti-ATG12. (**b**) HUVECs were stimulated with Aβ_1–40_ peptides for 24 h and the levels of conjugation of ATG12 and ATG5 were analyzed by immunoblotting. The cropped blot is displayed in the main figure, and its full-length blot is presented in Supplementary Fig. 1. Tubulin was used as a loading control. (**c**,**d**) Bar graph indicates the relative expression ratio of ATG5-ATG12 normalized to tubulin. Data shown are mean ± SEM of three independent experiments and were analyzed using Student’s *t*-test (*p < 0.05, **p < 0.01).

Several lines of evidence demonstrate that autophagic activation is involved in Aβ clearance and might play a role in the pathogenesis of AD. Since conjugation of ATG5-ATG12 is critical for the formation of autophagosome, we first asked whether conjugation of ATG5 and ATG12 is induced by Aβ. Western blot in primary rat cortical neurons and endothelial cells treated with Aβ, demonstrated that the conjugation between ATG5-ATG12 was increased (Fig. 1).

**Figure 2:**
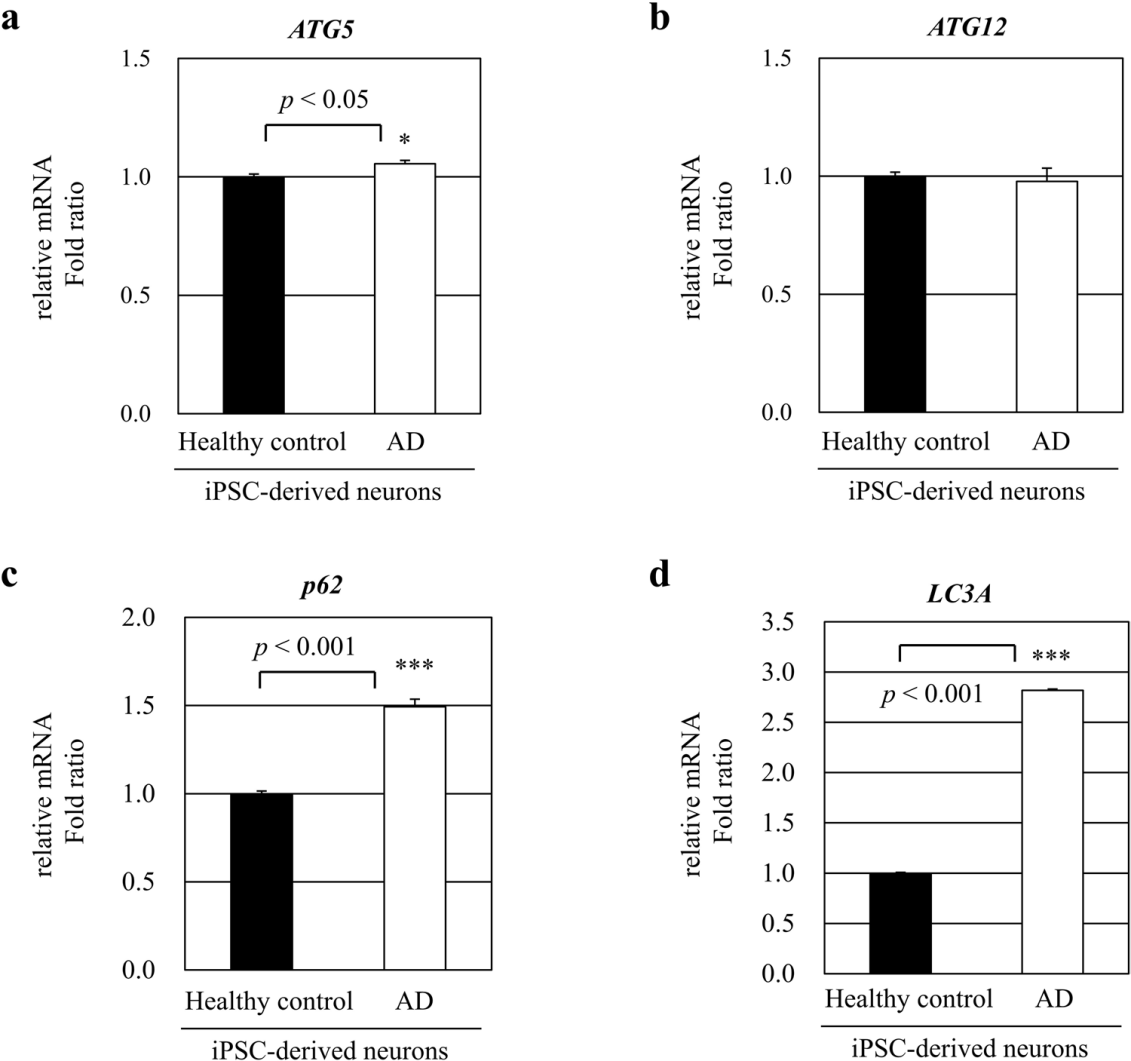
*ATG5* and *ATG12* mRNA expression in human iPSC-derived neuronal cells. Relative *ATG5*, *ATG12*, *p62*, and *LC3A* mRNA expression levels were analyzed in human iPSC-derived neural progenitor stem cells isolated from AD patient and healthy control donor (n = 3). Human iPSCs were differentiated into neurons in neuronal differentiation media. (**a**,**b**) *ATG5* and *ATG12* mRNA expressions were not changed in AD patient-derived iPSCs. (**c**,**d**) *p62* and *LC3A* mRNA expressions were significantly increased in human iPSC-derived neurons of an AD patient. Data shown are mean ± SEM of three independent experiments (*p < 0.05, ***p < 0.001).

Given that the protein levels of ATG5 and ATG12 are not altered in the brains of AD patients^12^, we sought to examine *ATG5* and *ATG12* mRNA levels in human induced pluripotent stem cell (iPSC)-derived neural progenitor stem cells isolated from a patient with AD. The mRNA levels of *ATG5* and *ATG12* were found unchanged in iPSC-derived neurons of an AD patient compared with those in iPSC-derived neurons of a healthy control donor (Fig. 2A,B). However, the mRNA levels of *p62* and *LC3A* were significantly increased in iPSC-derived neurons of an AD patient compared with those in iPSC-derived neurons of a healthy control donor (Fig. 2C,D).

**Figure 3:**
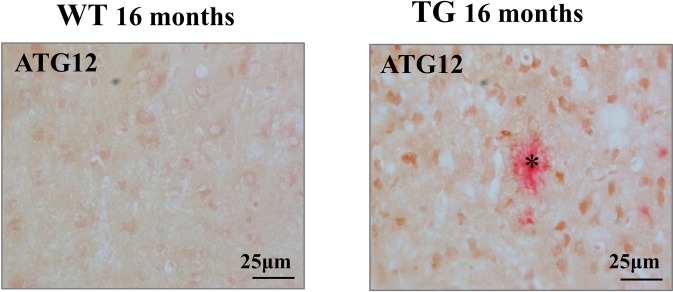
Immunostaining for ATG12 in the brain of APP transgenic mice. Brain cortex sections from 16-month-old wild type (WT) and APP transgenic (TG) mice were immunostained with anti-ATG12, and counterstained with Congo Red for amyloid plaques. Congophilic plaque was indicated by an asterisk.

Furthermore, the immunoreactivity of ATG12 was increased in the cortex of APPsw/PS1ΔE9 transgenic (APP Tg) mice as shown by diaminobenzidine staining in Fig. 3. Indeed, some of the ATG12-immunopositive cells were found at higher densities near the amyloid plaques stained by Congo Red in the brains of APP Tg mice. Mice lacking *ATG5* develop progressive deficits in motor function. Moreover, the autophagic flux in CA1 hippocampal neurons of AD patients was impaired with neuritic dystrophy^13,14^.

### Plasma ATG5 levels are elevated in AD patients

**Figure 4:**
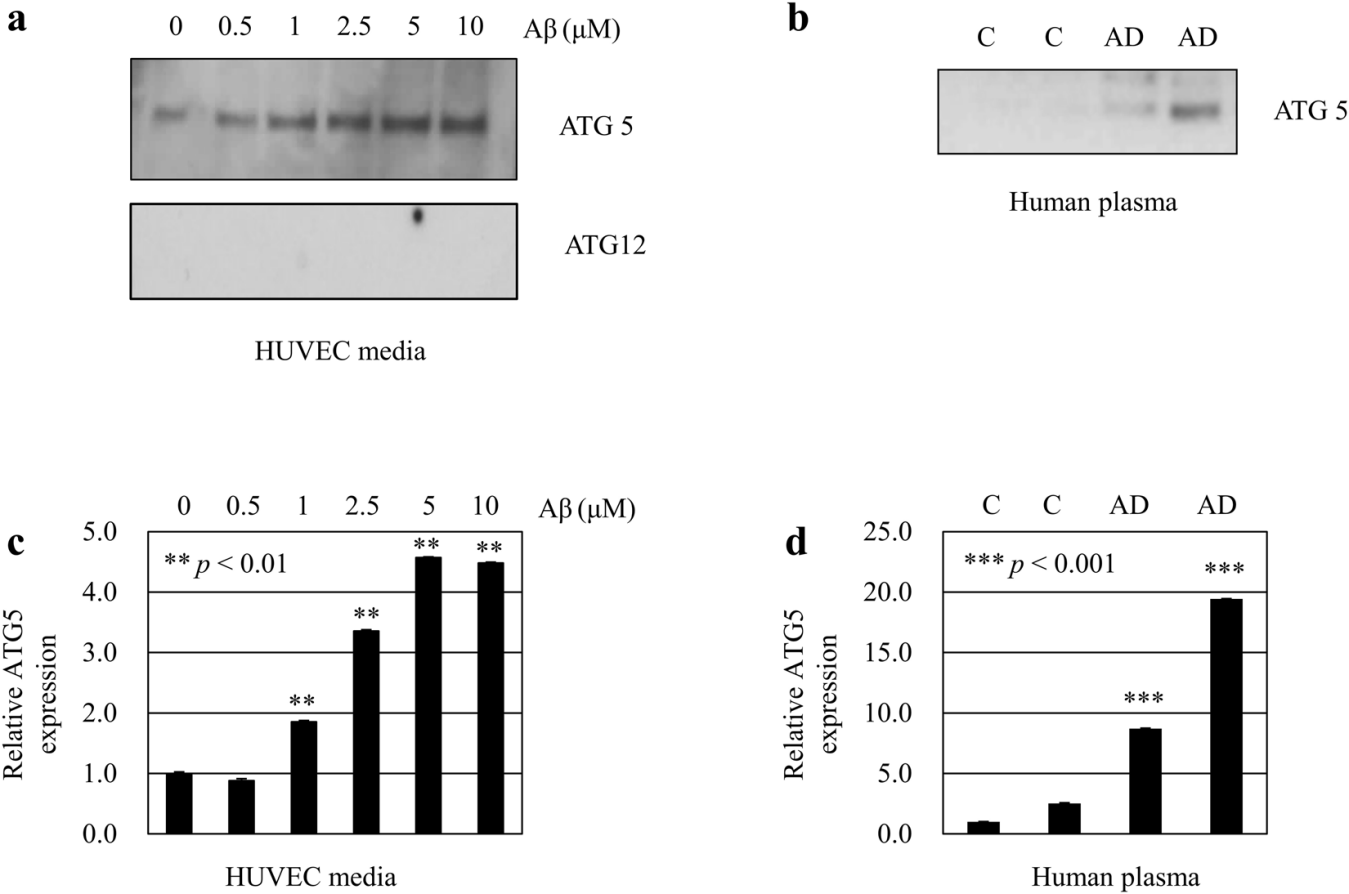
Secretion of ATG5 and ATG12 in cell-conditioned media and human plasma. Secreted ATG5 and ATG12 levels were analyzed by western blotting. (**a**) Conditioned media were harvested from HUVECs treated with 0~10 μM Aβ_1–40_ for 24 h. (**b**) Human plasma was collected from normal control participants and dementia patients. The cropped blots are displayed in the main figures, and its full-length blots are presented in Supplementary Fig. 2. (**c**,**d**) Bar graph indicates the relative expression ratio of ATG5 to control (not treated with Aβ). Data shown are mean ± SEM of three independent experiments and were analyzed using Student’s *t*-test (**p < 0.01, ***p < 0.001).

Recent studies have shown increased plasma level of autophagic markers in patients with diseases such as stroke^11^. For a more specific indication of the implication of autophagy in AD pathogenesis, we measured ATG5 and ATG12 levels in the plasma from patients with AD. Before that, we asked whether ATG5 and ATG12 were secreted into the conditioned medium from cells treated with Aβ. After treatment of Aβ in human umbilical vein endothelial cells (HUVECs) with Aβ, we found that ATG5 levels in the conditioned medium were increased (Fig. 4). This effect was dose dependent. However, we could not detect ATG12 band in the conditioned medium by western blot analysis.

Next, to clarify whether ATG5 and ATG12 levels are related to clinically overt dementia, we examined ATG5 and ATG12 plasma levels in mild cognitive impairment (MCI) and dementia patients as well as in control subjects without dementia. Table 1 presents the characteristics of the participants. The mean ages of patients with dementia were 75.3 ± 0.71 and 73.59 ± 0.54 years for patients with MCI and 72.0 ± 0.4 years for normal control participants; 75.3% of patients with dementia, 58.5% of patients with MCI, and 59% of control subjects were women.

**Table 1:** Baseline characteristics of the population.

Features	Control	MCI	Dementia	*p*-value
N (Male/Female)	127(52/75)	82(34/48)	69(17/52)	
Age (yr)	72.0 ± 0.4	73.59 ± 0.54	75.3 ± 0.71	<0.049
Education	9.03 ± 0.44	6.20 ± 0.53	3.71 ± 0.54	<0.001
MMSE	27.25 ± 0.18	24.74 ± 0.35	15.81 ± 0.69	<0.001
CDR	0.043 ± 0.01	0.26 ± 0.02	1.14 ± 0.09	<0.001
Total CHOL	195.2 ± 3.1	188.7 ± 3.6	203.2 ± 4.3	0.074
TG	137.1 ± 6.8	131.0 ± 7.2	152.9 ± 11.0	0.292
HDL	43.4 ± 0.8	43.0 ± 1.3	44.8 ± 1.1	0.236

Values are mean ± SEM.

Key: MCI, mild cognitive impairment; MMSE, Mini-Mental State Examination; CDR, clinical dementia rating; SEM, standard error of the mean.

Western blot analysis indicated that plasma ATG5 levels were increased in the dementia patients (n = 2) compared with controls without dementia (n = 2) (Fig. 4). For the analysis of ATG5 and ATG12 levels in the plasma, we established a sensitive ELISA method. Plasma ATG12 and ATG5 levels showed significant group difference between control and patients with dementia and MCI by Kruskal-Wallis test (*p* = 0.019, *p* = 0.023, respectively; Table 2). Plasma ATG12 levels were lower in the patients with MCI (17.2 ± 0.61 ng/mL) than the control subjects (19.85 ± 0.62 ng/mL) (*p* = 0.004). Plasma levels of ATG5 were significantly elevated in patients with dementia (149.3 ± 7.5 ng/mL) or MCI (152.9 ± 6.9 ng/mL) compared with the control subjects (129.0 ± 4.1 ng/mL) by Mann-Whitney U-test (*p* = 0.034, *p* = 0.016, respectively).

**Table 2:** Analysis of plasma ATG5 and ATG12 levels.

Features	Control	MCI	Dementia	*p*-value
N (Male/Female)	127(52/75)	82(34/48)	69(17/52)	
ATG12 (ng/ml)	19.85 ± 0.62	17.2 ± 0.61^a^	18.73 ± 0.83	**0.019**
ATG5 (ng/ml)	129.0 ± 4.1	152.9 ± 6.9^b^	149.3 ± 7.5^c^	**0.023**

Values are mean ± SEM.

^a^Compared with control; *p* = 0.004, ^b^compared with control; *p* = 0.016, ^c^compared with control; *p* = 0.034.

Table 3 shows that the plasma ATG12 levels were correlated with ATG5 plasma levels (*r* = 0.24, *p* < 0.001). Moreover, the overall clinical dementia rating (CDR) scores were correlated with ATG5 plasma levels (*r* = 0.124, *p* = 0.03). Interestingly, ATG5 and ATG12 levels were correlated with total cholesterol (TC) (*r* = −0.146, *p* = 0.015 and *r* = −0.17, *p* = 0.005, respectively).

**Table 3:** Correlation between plasma biomarkers and clinical rating scales.

Features	ATG12		ATG5	
	rho	*p*-value	rho	*p*-value
MMSE	0.05	0.35	−0.06	0.32
CDR	−0.03	0.57	0.124	**0**.**03**
ATG12			0.24	**<0**.**001**
ATG5	0.24	**<0**.**001**		
TC	−0.17	**0**.**005**	−0.146	**0**.**015**
TG	−0.022	0.71	0	1
HDL	−0.125	**0**.**038**	−0.06	0.25

Spearman rank correlation coefficient test was used for assessment of correlation.

## Discussion

It has been well demonstrated that autophagy is linked to neuronal apoptosis and is impaired in neurodegenerative diseases including AD, Parkinson’s disease, and Huntington’s disease^3,15^. Here, we showed that plasma ATG5 levels were increased both in APP Tg mice and human patients with dementia or MCI compared to healthy control subjects. To the best of our knowledge, this is the first study to determine the autophagic markers ATG5 and ATG12 in plasma from patients with AD.

Increasing evidences have shown that autophagy dysfunction is a common feature in AD. During AD progression, one of the earliest pathological features is the accumulation of autophagosomes^3^. It is well established that in neurodegenerative diseases, abnormal protein degradation through dysregulation of autophagic flux may result in neuronal loss. Indeed, fronto-temporal dementia and amyotrophic lateral sclerosis (ALS) have been linked to mutations in the charged multivesicular body protein-2B^16^. Moreover, polyglutamine aggregates in Huntington’s disease have been demonstrated to disrupt autophagy^17^ and decreased Beclin-1 levels were observed in sporadic AD^18^. However, the levels of protein components of the autophagy pathway such as ATG5, ATG12 and ATG7 in the brain were not significantly different between controls and AD patients^12^. On the other hand, ATG12-immunoreactive endothelial cells were found spatially associated with Aβ-positive plaques^8^. Consistently, our previous study showed that colocalization of ATG12 and small ubiquitin-related modifier 1 (SUMO1) was increased in the brain of an AD mouse model^19^. Furthermore, Aβ treatment increased conjugation of ATG5-ATG12 in human brain neuroglioma H4 cells and microglia^5,7^. In congruence with these observations, in our current study increased conjugation of ATG5-ATG12 induced by Aβ was also observed in primary rat neurons and endothelial cells, suggesting critical roles of autophagy in AD.

This is the first study to determine the levels of autophagic markers ATG5 and ATG12 in endothelial cell medium and in plasma from patients with AD. During disease progression, several proteins are released in the cerebrospinal fluid (CSF) and blood due of brain damage. In the central nervous system (CNS), autophagy can be activated by aging, nutrient deprivation and cerebral ischemia^20^. Recently, a study reported increase in autophagic markers such as LC3B and Beclin-1 in the CSF and peripheral blood in patients with acute ischemic stroke^11^. Moreover, plasma ATG5 levels were higher in the patients with coronary artery disease compared with healthy controls^9^. However, plasma ATG5 levels were lower in cerebral palsy patients compared with controls^10^. Yet, little is known about the regulation of autophagy pathways including autophagosome synthesis and autophagic flux in the blood of patients with AD. In the present study, we investigated alterations in ATG12 and ATG5 in the plasma of AD patients, triggered by the observed increased ATG5-ATG12 conjugation upon Aβ treatments *in vitro* (Fig. 1).

Our results showed that both LC3 and p62 mRNA levels were increased in human iPSC-derived neuronal cells (Fig. 2C,D). In addition, we confirmed that the levels of LC3 and p62 proteins in endothelial cells cultured in the presence of Aβ, pretreated with bafilomycin A_1_, were even more increased (data not shown). These results imply that Aβ treatment caused accumulation of autophagosomes and increased functional autophagic activity. In consistency with our paradoxical results, a previous study reported that the expression levels of p62 do not always inversely correlate with autophagic activity^21^. Indeed, until now there is no single “gold standard” in measuring autophagic flux. The p62 protein levels correlates with both activation and suppression of autophagy^22^. The autophagic system is highly dynamic and consists of a sequence of steps, each characterized by its own rate^23^. In this study, Aβ was demonstrated to increase autophagic activity and could also affect the autophagic steps, altering the rate of protein degradation through the entire autophagic pathway.

In addition, ATG5 levels after Aβ treatment were increased in the endothelial cell medium (Fig. 4). Plasma ATG5 levels were also increased in patients with AD (Table 2). This is a very interesting observation because an autophagic biomarker, ATG5, may be a potent candidate as a blood biomarker to reflect autophagy alterations in AD.

In conclusion, our data confirmed that the autophagic marker ATG5 is associated with AD pathophysiology. Additionally, we observed that plasma ATG5 levels in patients with AD were increased and associated with CDR, suggesting ATG5 as a potential biomarker in those at risk for AD.

## Methods

### Subjects

The obtained subjects used in this study were selected from the population-based Ansan Geriatric (AGE) cohort, Korea^24–26^. Dementia and MCI diagnoses were established by a Korean version of Consortium to Establish a Registry for Alzheimer’s Disease (CERAD-K) neuropsychological battery^27^. All participants were clinically assessed according to published guidelines, and each dementia patient met the criteria for the Diagnostic and Statistical Manual of Mental Disorders, fourth edition^28^. All dementia patients met the criteria for probable AD established by the National Institute of Neurological and Communicative Disorders and Stroke and the Alzheimer’s Disease and Related Disorders Association (NINCDS–ADRDA)^29^. Diagnosis of MCI was based on the Mayo Clinic criteria^24^ as described previously^30,31^. In total, blood samples from 278 subjects were collected, and the distribution on each of the subjects are shown in Table 1. Global clinical dementia rating (CDR) scores are 0 for normal, 2 and 3 for moderate to severe dementia^32^. All participants provided written informed consent and the study has been approved by the Institutional Review Board (IRB) of the Korea Centers for Disease Control and Prevention (KCDC). All experiments were conducted in accordance with relevant guidelines and regulations.

### Cell cultures

Human umbilical vein endothelial cells (HUVECs) purchased from Lonza (Walkersville, MD, USA) and cultured in Endothelial Growth Medium-2 (EGM-2)-MV BulletKit (Lonza). HUVECs were used at passages 5 to 9 for experimentation. Human iPSC-derived neural progenitor stem cells were purchased from Axol Bioscience (Little Chesterford, UK) and were differentiated to cerebral cortical neurons following the recommended manufacturer’s protocol^26^. Primary cortical neuronal cells obtained from cortexes of embryonic rat brains were maintained in neurobasal medium supplemented with B27 (Invitrogen) as described previously^33^.

### Antibodies and Reagents

Anti-ATG5 and anti-ATG12 were purchased from Cell Signaling Technology (MA, USA) and anti-tubulin was from Millipore (MA, USA). Synthetic Aβ_1–40_ peptides (Invitrogen, Camarillo, CA, USA) were dissolved in 1, 1, 1, 3, 3, 3-hexafluoro-2-propanol (Sigma, Saint Louis, MO, USA) and sequentially lyophilized. Lyophilized peptide was redissolved in dimethylsulfoxide (DMSO).

### Animals

APPsw/PS1ΔE9 transgenic mice were used for present study, as previously reported^19^. All animal experiments were performed in compliance with the guidelines for the care and use of laboratory animals by the Korea Centers for Disease Control and Prevention (KCDC) and approved by the Institutional Animal Care and Use Committee (IACUC) of the KCDC.

### Measurement of autophagic markers by ELISA

All the cell-free plasma samples were aliquoted and stored at −80 °C until assayed collectively for ATG5 and ATG12 by an investigator who was blinded to patient assignment. The levels of ATG5 and ATG12 were determined by the enzyme-linked immunosorbent assays kit following the manufacturer’s instructions (USCN, Wuhan, China).

### Western blotting

Cells and mouse cortex regions were lysed in radio-immunoprecipitation assay buffer (RIPA buffer; 20 mM Tris, pH 7.4, 150 mM NaCl, 1 mM Na_3_VO_4_, 10 mM NaF, 1 mM EDTA, 1 mM EGTA, 0.2 mM PMSF, 1% Triton X-100, 0.1% SDS, 0.5% deoxycholate), protein concentrations were determined using a Bradford protein assay^19^. Equal amounts of lysates were separated by SDS-PAGE with bolt 4~12% Bis-Tris gradient gel in MES SDS buffer (Life technology, NY, USA). Proteins were transferred onto polyvinylidene difluoride membranes (Millipore, Billerica, MA) and blocked for 1 h at room temperature in 5% nonfat dry milk. Membranes were incubated with the anti-ATG5 (1:1,000), anti-ATG12 (1:1,000), and anti-tubulin (1:10,000).

### Real-time reverse transcription polymerase chain reaction

Real-time quantitative RT-PCR analysis was performed using SYBR Green two-step qRT-PCR kit (Applied Biosystems, Warrington, UK). PCR fragments were amplified for 10 min at 95 °C, followed by 40 cycles for 15 seconds at 95 °C and 1 min at 58 °C. The following primers were used: *ATG5* sense 5′-GGCCATCAATCGGAAACTCA-3′ and antisense 5′-ACAGGACGAAACAGCTTCTG-3′; *ATG12* sense 5′-TGTGTTGCAGCTTCCTACTTCA-3′ and antisense 5′-TCAATGAGTCCTTGGATGGTTC-3′; *p62* sense 5′-TCCAGTCCCTACAGATGCCA-3′ and antisense 5′-GAGAGGGACTCAATCAGCCG-3′; *LC3A* sense 5′-CGCTACAAGGGTGAGAAGCA-3′ and antisense 5′-TTCACCAGCAGGAAGAAGGC-3′. RT-qPCR analysis were performed on QuantStudio 6 Felx Real-Time PCR System (Applied Biosystems, Warrington, UK). Ct values of the control and stimulated sample were calculated and the transcript levels were analyzed 2^−ΔΔCt^ method and normalized to the reference gene levels. All the RT-qPCR reaction were performed as triplicates.

### Immunohistochemistry and Congo Red Staining

Brains from 16-month-old APP Swedish/PS1dE9 transgenic (Tg) mice together with their wild-type controls were fixed in 4% (w/v) paraformaldehyde. Cryostat sagital sections were cut on a sliding microtome into 10 μm slices at −20 °C and placed on a microslide for immunostaining. The cortex sections were immunostained with rabbit monoclonal antibodies against ATG12 (1:100, Cell Signaling Technology, 4180). When required, immunolabeled sections were then incubated for 3 min in a solution of 20% Congo Red (Sigma, C6277). Axiolab Pol Polarizing microscope (Carl Zeiss, Jena, Germany) with AxioVision Release 4.8 software was used for analysis of 3, 3′-diaminobenzidine photomicrographs.

### Statistical analyses

The results were presented as mean ± standard error of the mean (SEM). Kruskal-Wallis test and Mann-Whitney U-test were performed to analyze demographic and plasma levels of target proteins between dementia, MCI and control groups. Correlation between factors was checked by Spearman’s method. Statistical analyses of the present study were performed using SPSS 12.0 (IBM, NY, USA). Values of *p* < 0.05 were regarded as statistically significant.

## Supplementary information

Supplementary Information

## References

[1] Scheff SW, Price DA, Schmitt FA, Mufson EJ (2006). Hippocampal synaptic loss in early Alzheimer’s disease and mild cognitive impairment. Neurobiology of aging.

[2] Wang CW, Klionsky DJ (2003). The molecular mechanism of autophagy. Mol Med.

[3] Nixon RA (2005). Extensive involvement of autophagy in Alzheimer disease: an immuno-electron microscopy study. Journal of neuropathology and experimental neurology.

[4] Nixon RA, Yang DS (2011). Autophagy failure in Alzheimer’s disease–locating the primary defect. Neurobiology of disease.

[5] Lipinski MM (2010). Genome-wide analysis reveals mechanisms modulating autophagy in normal brain aging and in Alzheimer’s disease. Proceedings of the National Academy of Sciences of the United States of America.

[6] Mizushima N, Yoshimori T, Ohsumi Y (2003). Role of the Apg12 conjugation system in mammalian autophagy. The international journal of biochemistry & cell biology.

[7] Cho MH (2014). Autophagy in microglia degrades extracellular beta-amyloid fibrils and regulates the NLRP3 inflammasome. Autophagy.

[8] Ma JF, Huang Y, Chen SD, Halliday G (2010). Immunohistochemical evidence for macroautophagy in neurones and endothelial cells in Alzheimer’s disease. Neuropathology and applied neurobiology.

[9] Demircan G, Kaplan O, Ozdas SB (2018). Role of autophagy in the progress of coronary total occlusion. Bratislavske lekarske listy.

[10] Xu J (2017). A Variant of the Autophagy-Related 5 Gene Is Associated with Child Cerebral Palsy. Frontiers in cellular neuroscience.

[11] Li H, Qiu S, Li X, Li M, Peng Y (2015). Autophagy biomarkers in CSF correlates with infarct size, clinical severity and neurological outcome in AIS patients. Journal of translational medicine.

[12] Crews L (2010). Selective molecular alterations in the autophagy pathway in patients with Lewy body disease and in models of alpha-synucleinopathy. PloS one.

[13] Bordi M (2016). Autophagy flux in CA1 neurons of Alzheimer hippocampus: Increased induction overburdens failing lysosomes to propel neuritic dystrophy. Autophagy.

[14] Hara T (2006). Suppression of basal autophagy in neural cells causes neurodegenerative disease in mice. Nature.

[15] Bahr BA, Bendiske J (2002). The neuropathogenic contributions of lysosomal dysfunction. Journal of neurochemistry.

[16] Skibinski G (2005). Mutations in the endosomal ESCRTIII-complex subunit CHMP2B in frontotemporal dementia. Nature genetics.

[17] Ravikumar B (2004). Inhibition of mTOR induces autophagy and reduces toxicity of polyglutamine expansions in fly and mouse models of Huntington disease. Nature genetics.

[18] Pickford F (2008). The autophagy-related protein beclin 1 shows reduced expression in early Alzheimer disease and regulates amyloid beta accumulation in mice. The Journal of clinical investigation.

[19] Cho SJ (2015). SUMO1 promotes Abeta production via the modulation of autophagy. Autophagy.

[20] Adhami F, Schloemer A, Kuan CY (2007). The roles of autophagy in cerebral ischemia. Autophagy.

[21] Sahani MH, Itakura E, Mizushima N (2014). Expression of the autophagy substrate SQSTM1/p62 is restored during prolonged starvation depending on transcriptional upregulation and autophagy-derived amino acids. Autophagy.

[22] Loos B, du Toit A, Hofmeyr JH (2014). Defining and measuring autophagosome flux-concept and reality. Autophagy.

[23] Zhang XJ, Chen S, Huang KX, Le WD (2013). Why should autophagic flux be assessed?. Acta pharmacologica Sinica.

[24] Petersen RC (1999). Mild cognitive impairment: clinical characterization and outcome. Archives of neurology.

[25] Han C, Jo SA, Kim NH, Jo I, Park MH (2009). Study design and methods of the Ansan Geriatric Study (AGE study). BMC neurology.

[26] Cho SJ, Park MH, Han C, Yoon K, Koh YH (2017). VEGFR2 alteration in Alzheimer’s disease. Scientific reports.

[27] Lee JH (2002). Development of the Korean version of the Consortium to Establish a Registry for Alzheimer’s Disease Assessment Packet (CERAD-K): clinical and neuropsychological assessment batteries. The journals of gerontology.

[28] Association, A. P. *Diagnostic and Statistical Manual of Mental Disorders*. 4th edition edn, (American Psychiatric Press, 1994).

[29] McKhann G (1984). Clinical diagnosis of Alzheimer’s disease: report of the NINCDS-ADRDA Work Group under the auspices of Department of Health and Human Services Task Force on Alzheimer’s Disease. Neurology.

[30] Kim J (2007). Plasma homocysteine is associated with the risk of mild cognitive impairment in an elderly Korean population. The Journal of nutrition.

[31] Jang BG (2010). Plasma carbonic anhydrase II protein is elevated in Alzheimer’s disease. Journal of Alzheimer’s disease: JAD.

[32] Morris JC (1993). The Clinical Dementia Rating (CDR): current version and scoring rules. Neurology.

[33] Yun SM (2013). SUMO1 modulates Abeta generation via BACE1 accumulation. Neurobiology of aging.

