# Supplementary material for: Plasma ATG5 is increased in Alzheimer’s disease
Source: Sci Rep. 2019 Mar 18;9:4741. doi: 10.1038/s41598-019-41347-2 (PMC6427023; doi:10.1038/s41598-019-41347-2)

**Supplementary Information**

**Plasma ATG5 is increased in Alzheimer’s disease**

Sun-Jung Cho^1, §^, Hyun Joung Lim^1, §^, Chulman Jo^1^, Moon Ho Park^2^, Changsu Han^3^ and Young Ho Koh^1,*^

^1^ Division of Brain Diseases, Center for Biomedical Sciences, Korea National Institute of Health, 187 Osongsaengmyeong2-ro, Osong-eup, Heungdeok-gu, Cheongju-si, Chungcheongbuk-do 28159, Korea. ^2^ Departments of Neurology and ^3^ Psychiatry, College of Medicine, Korea University, Ansan Hospital, 123 Jeokgeum-ro, Danwon-gu, Ansan-si, Gyeonggi-do 15355, Korea.

*Correspondence and requests for materials should be addressed to Y.H.K (email: [kohyoungho122@gmail.com](mailto:kohyoungho122@gmail.com)).

^§^Sun-Jung Cho and Hyun Joung Lim contributed equally to this work.

**Supplementary Figures**

**Supplementary Figure 1. Full blot of Fig. 1a, 1b**

**
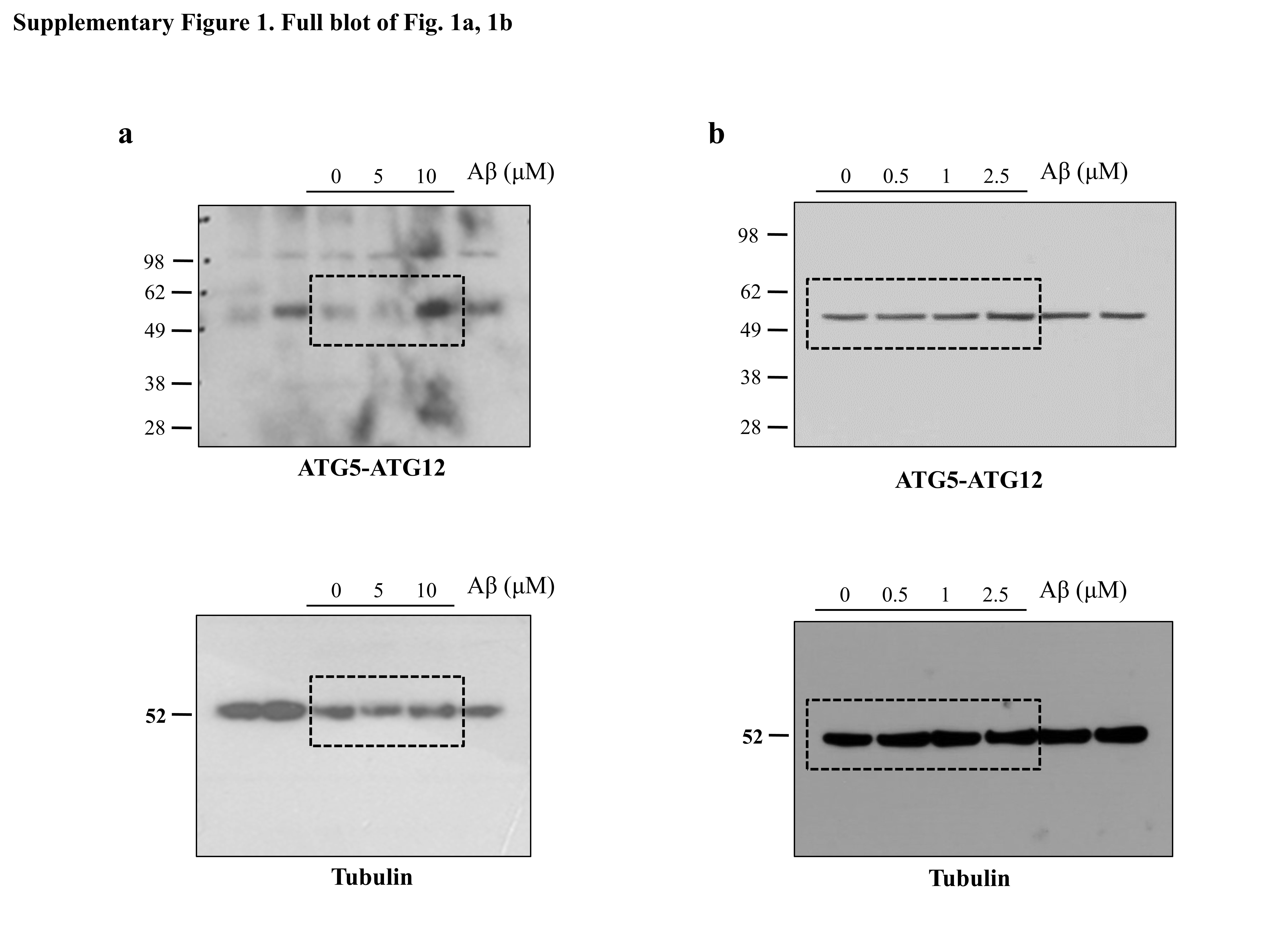
**

**Supplementary Figure 2. Full blots of Fig. 4a, 4b**


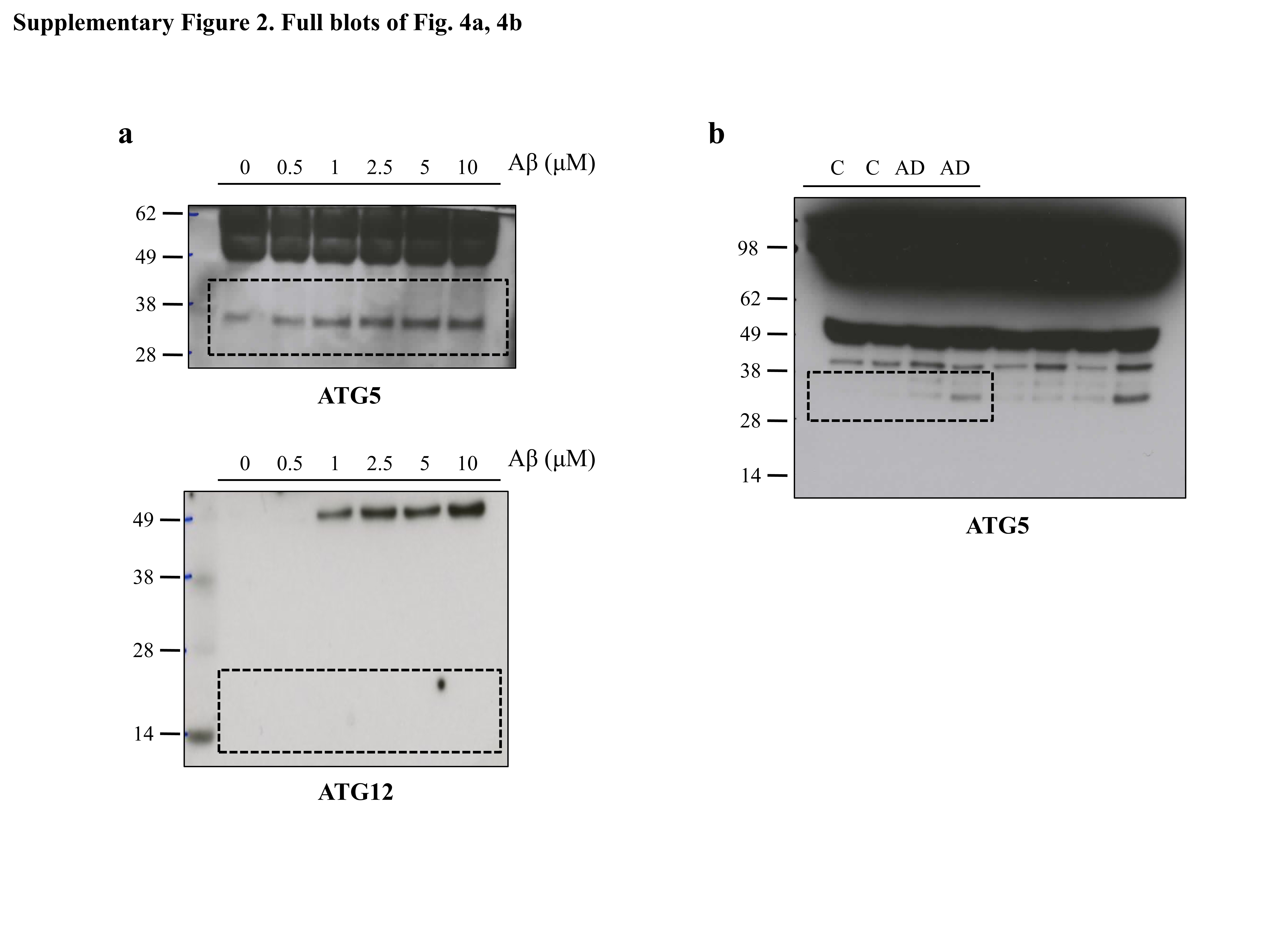

Supplement: Supplementary file 1 — Supplementary Information [file 41598_2019_41347_MOESM1_ESM.doc]
